# Graft versus host disease and microchimerism in a *JAK3 deficient* patient

**DOI:** 10.1186/s13223-019-0361-2

**Published:** 2019-08-22

**Authors:** Zahra Shahbazi, Nima Parvaneh, Shirin Shahbazi, Hamzeh Rahimi, Mohammad Hamid, Davoud Shahbazi, Samaneh Delavari, Hassan Abolhassani, Asghar Aghamohammadi, Reza Mahdian

**Affiliations:** 1 Molecular Medicine Department, Pasteur Institute of Iran, Pasteur St., 12 Farvardin Ave., Tehran, 1316943551 Iran; 2 Research Center for Immunodeficiencies, Pediatrics Center of Excellence, Children’s Medical Center, Tehran, Iran; 3 Department of Medical Genetics, Faculty of Medical Sciences, Tarbiat Modares University, Tehran, Iran; 4 Shahid Hoseini School, Department of Education, Semirom, Isfahan Iran; 5 Division of Clinical Immunology, Department of Laboratory Medicine, Karolinska Institute at Karolinska University Hospital Huddinge, Stockholm, Sweden; 6 Department of Pediatrics, Children Medical Center Hospital, Tehran University of Medical Science, Tehran, Iran

**Keywords:** Severe combined immunodeficiency, Graft versus host disease, Microchimerism, *JAK3* deficiency, Short Tandem Repeat

## Abstract

**Background:**

The lymphohematopoietic cells originating from feto-maternal trafficking during pregnancy may cause microchimerism and lead to materno-fetal graft versus host disease (GVHD) in severe combined immunodeficiency (SCID) patients. However, definitive diagnosis between GVHD and Omenn’s syndrome is often difficult based on clinical and immunological phenotypes particularly in the patients with hypomorphic mutations.

**Case presentation:**

A 3-year-old girl with a history of erythroderma and immunodeficiency was studied. The whole exome sequencing method was used to find the disease-causing variants, and T-A cloning and Quantitative Florescence Polymerase Chain Reaction (QF-PCR) methods were utilized to detect the presence of mosaicism or microchimerism. We identified a homozygous missense Janus Kinase 3 mutation (*JAK3*, c.2324G>A, p.R775H) as a new disease-causing variant in the patient, and the presence of microchimerism with maternal origin was proven as an underlying cause of her clinical presentation.

**Conclusion:**

The findings highlighted the importance of appropriate diagnostic approach in GVHD cases with hypomorphic *JAK3* mutations. When analyzing the results of the next generation sequencing, the possibility of microchimerism should be considered based on the context of the disease.

## Background

The clinical differential diagnosis of erythroderma associated with immunodeficiency and failure to thrive (FTT) in neonates includes Omenn’s syndrome (OS) and graft versus host disease (GVHD) in patients with severe combined immunodeficiency (SCID). OS is a rare, autosomal recessive or X-linked disorder in infancy caused by atypical mutations, particularly missense variants, of the recombination activating gene 1 and 2 (*RAG1* and *RAG2*). OS rarely results from mutations in *ARTEMIS*, *IL7RA*, *RMRP*, *ADA*, *DNA LIG4*, *IL2RG*, *AK2*, or can be associated with the DiGeorge syndrome; however, some cases have no defined gene mutation. Interestingly, siblings with identical mutation have developed either OS or SCID, suggesting that modifier genetic or environmental factors affect the clinical/immunologic phenotype. It has been suggested that in OS patients early infections may lead to the expansion of poorly reactive autologous oligoclonal T cells [1].

On the other hand, atypical SCID comprises a variety of immunologic profile of both T and B lymphocytes which may challenge the early diagnosis of patients with combined immunodeficiency. One specific feature of SCID patients, which sometimes can simulate the clinical presentation of OS, is GVHD because of the presence of alloreactive cells originating from transplacentally obtained maternal T lymphocytes [2–4]. Immunocompetent neonates with efficient T-cell immunity can quickly reject the human leucocyte antigen (HLA) mismatched maternal cells. In contrast, SCID newborns fail to remove these cells; so that maternal T cells have been detected in 24% to 40% of patients undergoing hematopoietic stem cell transplantation [2–4]. To differentiate between OS and GVHD in SCID patients, immunological investigations, pathological examination of skin biopsies and molecular tests demonstrating the presence of mosaicism or microchimerism must be implemented. Mosaicism involves the presence of two or more populations of cells with different genotypes in an individual that has developed from a single fertilized egg. These cells are autologous cells and lead to OS in the SCID patients, which is the concern of our study. Chimerism is a condition whereby a person has not one but two complete genomes and two or more cell populations with distinct genotypes. These cells are allogeneic cells and may lead to GVHD in SCID patients. In terms of differential diagnosis, in a person with erythroderma associated with immunodeficiency and failure to thrive, evidence of micro-chimerism and autologous cells existence suggests that the person is not suffering from OS but confirms the maternal GVHD diagnosis [5, 6].

**Fig. 1:**
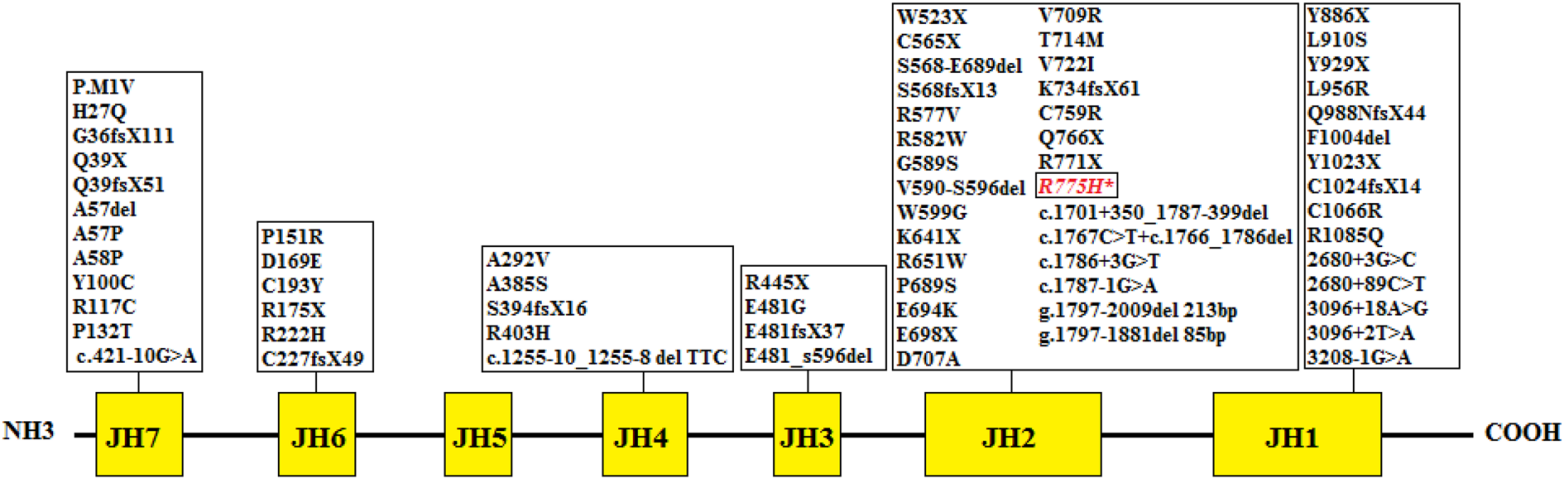
The diagram of JAK3 protein and mutations previously reported in its 7 JH domains. The mutation found in the current study is shown in a box fall into JH2 domain

In this report, we describe a JAK3 deficient SCID patient with a hypomorphic missense mutation. Whole exome sequencing (WES) and confirmatory tests were used for the accurate molecular diagnosis of a microchimeric in this patient. Since JAK3 protein regulates the growth and maturation of T-cell lymphocytes and natural killer cells and it is also important for B-lymphocytes maturation, mutations in this gene result in the absence of T cells and natural killer cells and a normal number of poorly functioning B cells. The affected infants typically develop chronic diarrhea, a fungal infection in the mouth called oral thrush, pneumonia, skin rashes and FTT. JAK3 deficient SCID patients usually live only into early childhood. The diagram related to the JAK3 protein and the different JH domains and mutations reported to exist in this protein so far is presented in Fig. 1.

## Case presentation

### Clinical evaluation and immunological assays

Informed consent of the patient’s parents was obtained for performing the studies in accordance with the principles of the ethics committee of the Tehran University of Medical Sciences. An evaluation sheet was used to summarize demographic information related to the patient registered in the national registry [7, 8] including name, gender, date of birth, age of onset of symptoms, clinical symptoms, age at diagnosis, family history and consanguinity, previous history of medications and vaccination, and laboratory and molecular data. Pathological examination of skin biopsy sample of the patient and advanced immunological assays were carried out to confirm the diagnosis. The diagnosis of SCID was established on the standard criteria introduced by the ESID and Pan-American Group for Immunodeficiency (http://esid.org/Working-Parties/Registry/Diagnosis-criteria) [8]. The medical severity phenotype of SCID patients was defined by having 2 of the following criteria: early age at onset of symptoms (< 1 month), mortality (< 1 year), absent CD3+ or CD4+ or CD8 T+ cells, development of opportunistic infections, and development of severe infectious complications during the course of the disease (sepsis, central nervous system infections, osteomyelitis, and invasive bacterial infection).

Early immunological screening was performed, which included complete blood count, measurement of immunoglobulin (IgG, IgA, IgM and IgE) serum levels using the nephelometry technique, and lymphocyte counting, immunophenotype analysis using flow cytometry (CD3, CD4, CD8, CD19 and CD16/56) and proliferation response of lymphocyte to Phytohemagglutinin (PHA). Details of other immunologic tests have been described previously [9].

### Genetic evaluation and confirmatory sequencing

In order to identify the genes causing OS or SCID, a DNA sample of the patient was sequenced in the Macrogen company (South Korea) using WES on the Illumina HiSeq platform and analysis was performed based on the previously published pipeline [8]. The pathogenicity of the identified disease attributable gene variants was re-evaluated using the updated guideline for interpretation of molecular sequencing by the American College of Medical Genetics and Genomics (ACMG), considering allele frequency in the population database, computational data, immunologic/functional data, familial segregation and parental data and clinical phenotyping [8]. Polymerase chain reaction (PCR) and Sanger-sequencing method was used on the patient’s DNA sample (the primers sequences are available in Additional file 1: Table S1) and those of the relatives.

### TA-cloning and Quantitative Florescence Polymerase Chain Reaction (QF-PCR)

T-A cloning of PCR fragments in the pGEM vector was performed, as described previously [10], to evaluate the probability of masaicism or microchimerism. We used *E. coli* Top10 as host cells, and tetracycline resistance and ampicillin resistance as a selection marker, and vector transformation marker, respectively. The recombinant plasmids were extracted and sent to the Macrogen company (South Korea) for Sanger sequencing (Fig. 1b, more details are available in Additional file 2). In order to determine the origin of the different lymphocyte lines in the patient, we used QF-PCR assay for trisomy Short Tandem Repeat (STR)-markers (Kawsar Biotechnology company, Iran) on Applied Biosystem Genetic Analyzer interpreted by using GeneMaper 4.1 software.

## Results

**Fig. 2:**
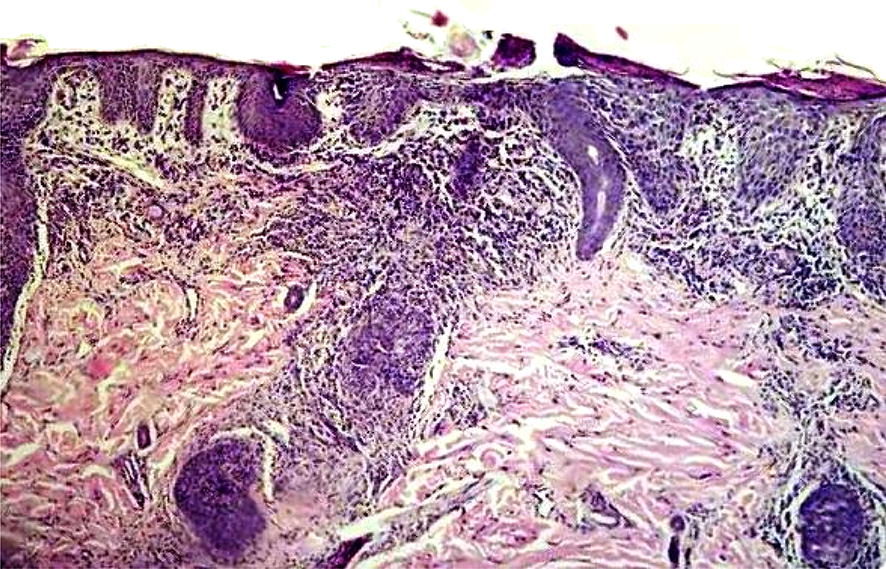
Skin biopsy of the patient show acanthosis and moderate to severe lymphocytic and histiocytic infiltration in upper and also hypodermis with vague granulomatous formation. Also granulation inflammation with presence of acid fast bacilli is visible (Ziehl–Neelsen staining)

The participant in our study included a 3-year-old Caucasian girl born out of a first-degree consanguineous marriage, with a history of diarrhea (without blood and microbiologic tests failed to show specific pathogens onset at age 3 months), FTT (weight drop down two major percentile lines at age 6 months) and oral thrush (at age 4 months, resolved with 1% clotrimazole solution). The child had respiratory distress (chest X-ray reported with nonspecific air bronchograms), wheezing, erythroderma (diffused involving 50% of the body’s surface with exfoliation and responded moderately to immunosuppressive drug), alopecia and skin dryness. There was no family history of SCID and she had an unaffected male sibling. She did not suffer from lymphadenopathy, hepatosplenomegaly, pneumonia, invasive infection, liver involvement, BCGosis and eosinophilia. The pathological examination of the skin biopsy at the age of 18 months revealed acute as well as chronic inflammation extending from the upper to the deep dermis (Fig. 2).

The patient’s white blood cell count was normal with a slightly higher proportion of lymphocytes. The percentage of lymphocyte subsets showed an increased proportion of CD4+ T cells (Table 1). She was found to have an immunoglobulin G (IgG) level of 332 mg/dL, IgA 5 mg/dL, but a normal serum level of IgM and IgE (87 mg/dL and 34.8 IU/mL, respectively).

**Table 1:** Differential frequency of Lymphocytes and serum levels of immunoglobulins in the patient

Parameters	Patient	Normal range^a^
WCB (cell*10^3^/µL)	10.3^b^	5–17
Eosinophils % (count, cell/mL)	8.2 (851)^b^	0–1
Platelets, count, cells*10^9^/L	325^b^	150–400
Hemoglobin, g/dL	10.4^b^	11.5–14.0
Lymphocyte % (count, cell/mL)	78.7 (8169)^b^	50–70
CD3% (count, cell/mL)	93.5 (7643)^b^	56–75
CD4% (count, cell/mL)	86.4 (7063)^b^	28–47
CD4+ CD45RA+ CD45RO− (naïve) % of helper T cells	59	11–53
CD4+ CD45RA-CD45RO+ (memory) % of helper T cells	28	31–74
CD4+ HLA-DR+ CD38− (activated) % of helper T cells	3	2–11
CD8% (count, cell/mL)	4.6 (382)^b^	16–30
CD8+ CD45RA+ CD45RO− (naïve) % of cytotoxic T cells	75	27–69
CD8+ CD45RA-CD45RO+ (memory) % of cytotoxic T cells	15	12–50
CD8+ HLA-DR+ CD38− (activated) % of % of cytotoxic T cells	2	2–22
CD16% (count, cell/mL)	4.2 (343.1)^b^	4–17
CD19% (count, cell/mL)	4.05 (331)^b^	14–33
Lymphocyte proliferation responses to PHA	1.5	> 0.3
Quantitative CD4+ PHA response (c/µL)	120^b^	170–3499
Quantitative CD8+ PHA response (c/µL)	25^b^	76–3640
IgG (mg/dL)	332^b^	295–1156
IgA (mg/dL)	5^b^	27–246
IgM (mg/dL)	87^b^	37–184
Anti-tetanus (IU/mL)	0.05^b^	> 0.1
Anti-diphtheria (IU/mL)	0.01^b^	> 0.1
TRECs	10^c^	23–67

*TRECs* T-cell receptor excision circles, *PHA* phytohemagglutinin

^a^The normal range of these quantities is derived from the http://www.palms.com.au

^b^ Measured at age 10 months

^c^Measured at age 3 years

**Fig. 3:**
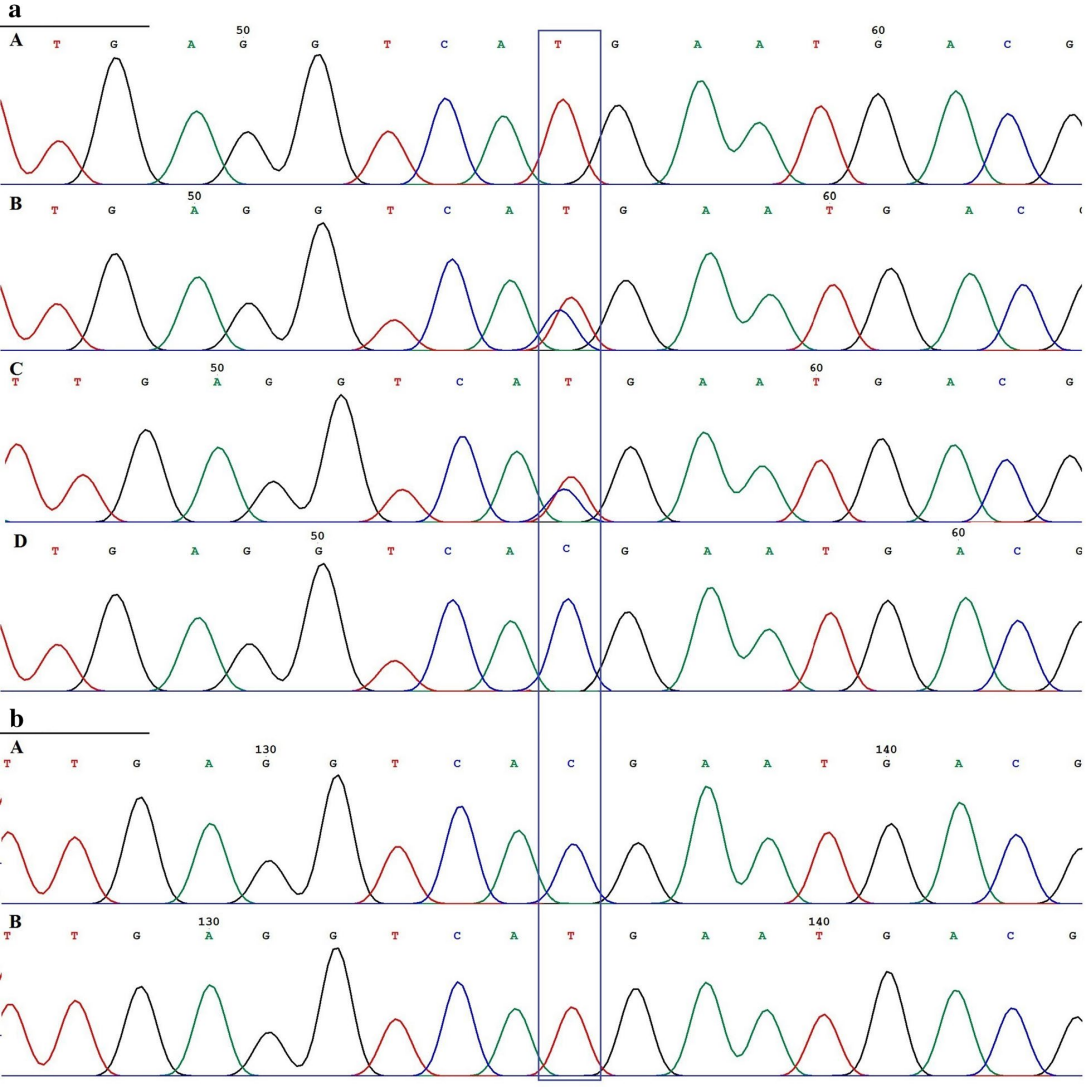
**a** *JAK3* gene Sanger sequencing result for the proband (A) shows homozygous genotype for mutant T allele, patient’s father (B) and her mother (C) are heterozygous, and a normal person (D) that is homozygous for wild type C allele. **b** Sanger sequencing result of the plasmids containing different *JAK3* gene alleles, (A) Wild type C allele, (B) Mutant T allele

**Fig. 4:**
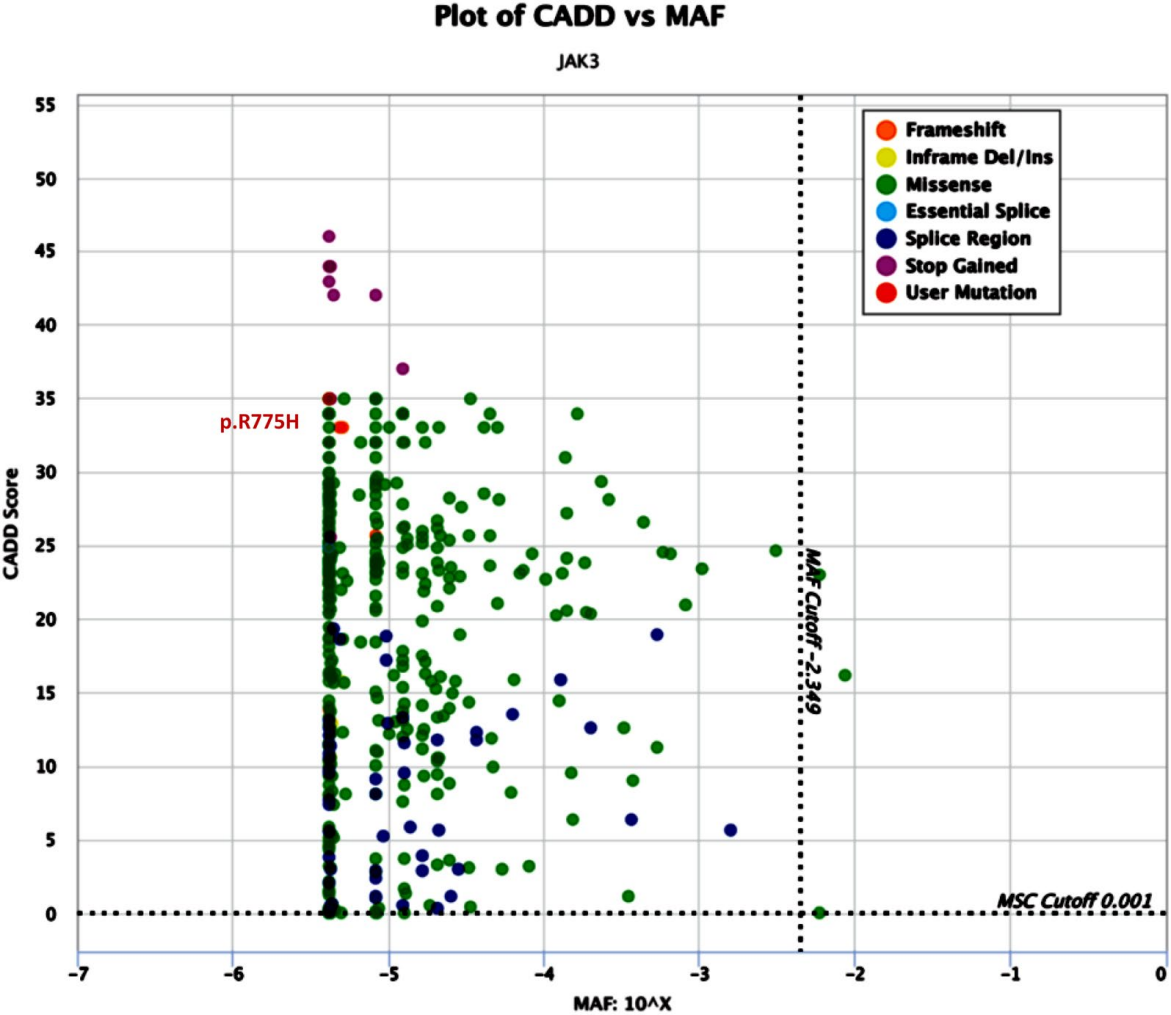
Correlating Combined Annotation Dependent Depletion (CADD) scores with minor allele frequencies (MAF) for pathogenic *JAK3* mutations in the proband and other variants (missense mutations, splice acceptor mutations, splice donor mutations, start losses and frameshift mutations) reported in the population databases (ExAC: http://exac.broadinstitute.org, n = 60,706 exomes and gnomAD: http://gnomad.broadinstitute.org, n = 123,136 exomes and 15,496 genomes). *MSC* mutation significance cutoff

Despite the normal count of T-cells, due to severity of medical presentation, proliferation response of lymphocyte to PHA was also tested and indicated a functional cellular defect. The targeted sequencing results related to OS associated genes (mainly in Iran due to *RAG1* and *RAG2* mutations) showed no pathogenic variants. However, by using WES analysis followed by confirmatory sequencing, we identified a homozygous missense variant in *JAK3* gene exon 17 (c.2324G>A, p.R775H) in the patient while her parents were heterozygous for the variant (Fig. 3a, Additional files 3, 4: Table S2). The CADD score with minor allele frequencies (MAF) for pathogenic *JAK3* mutations in the proband and other variants is presented in Fig. 4. This variant affects protein kinase 1 domain presenting a very low population allele frequency (3.9 × 10^−6^). Pathogenicity predictive scores (Table 2) indicated that this variant was probably deleterious. According to the ACMG variant classification guideline (https://www.acmg.net/*)*, this variant was assigned to pathogenic strong 2 (PS2) category.

**Table 2:** Interpretation scores for the pathogenicity of p.R775H variant in *JAK3* gene (neu: neutral; del: deleterious)

Software name	Predict SNP	PhD-SNP	Polyphen-1	Polyphen-2	SIFT	SNAP	Mutation taster prediction	Pathogenicity (ACMG)
Score/prediction	72% del	61% del	74% del	81% del	79% del	72% del	Disease causing	Likely Pathogenic

**Fig. 5:**
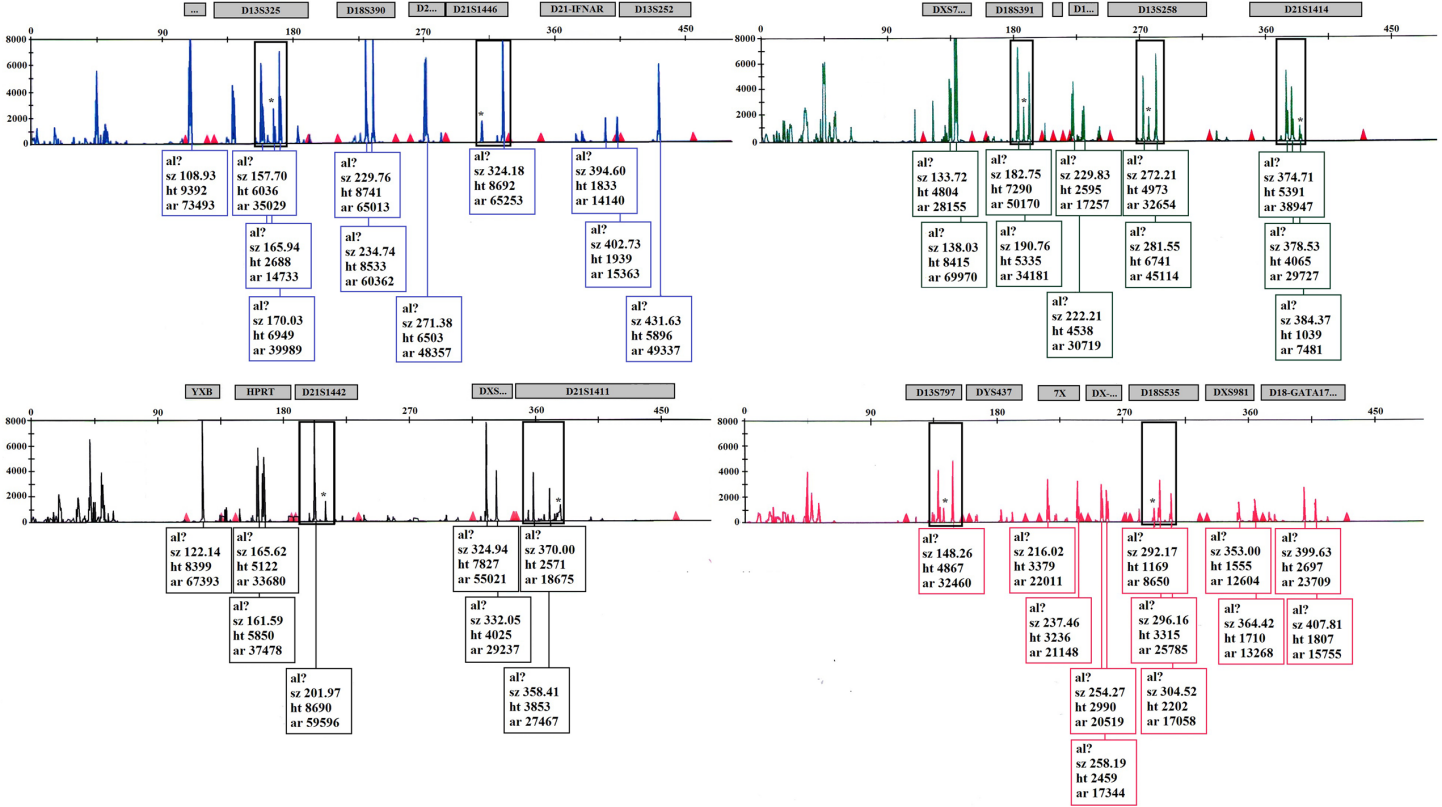
QF-PCR results for trisomy STR-marker indicating the presence of three different haplotypes for the investigated STR markers in the patient’s blood cells. In a normal person, for each of the STR markers shown in the figure, two peaks with an almost equal size (for two alleles in heterozygote genotype) or a single long peak (for one allele in homozygote genotype) are expected. In our patient as show with star sign (*), some of the markers have a short extra peak that is the maternal allele exists in maternal T cells but did not inherit to the child during fertilization. Moreover a skewed allele ratio due to the presence of the maternal allele that is common between mother and child is seen. This allele leads to an increase of the length of one of the binary peaks. These two finding suggest the presence of two maternal alleles in a low dosage. We have calculated the allele dosage ratios by dividing the larger peak area to the smaller one. There are no four-allele results. Fragment size is in bp on horizontal line, arbitrary fluorescence units shown on vertical axis. Alleles labeled according to the marker name

Interestingly, on visualization of the mutation region in the WES bam file, we observed the presence of wild type allele (G allele) suggesting the occurrence of mosaicism or microchimerism with an approximate frequency of 17% (Additional file 5: Figure S1). By sequencing of 10 plasmids containing the mutation-harboring fragments, we found one wild type allele (G allele) and nine mutant alleles (A allele) with an allelic ratio of 1 to 9 in the patient (Fig. 3b, Additional file 6: Figure S2, Additional file 7: Figure S3). Finally, QF-PCR results showed the presence of maternally originated lymphocytes in the patient’s blood (Fig. 5). Overall, the results of molecular analyses confirmed that maternally originated cells caused chimerism or GVHD and were responsible for the atypical SCID clinical manifestations of the proband. Patient is a candidate for Hematopoietic stem cell transplantation (HSCT) but yet an appropriate donor could not be identified.

## Discussion and conclusions

A commonly described abnormality in SCID patients is a type of microchimerism named graft-versus host disease (GVHD) caused by the prenatal or perinatal placental transmission of maternal T lymphocytes and the failure in SCID patients to recognize and reject foreign cells. By means of sensitive methods, this microchimerism was observed in up to 42% of healthy newborns’ cord blood samples, and incidence of maternal engraftment was reported in 24% to 40% of SCID patients. The maternal engraftment manifestations may vary from severe or mild GVHD to lack of this symptom. The basis of this variability is poorly understood. The proliferative properties of transfused maternal T cells may be modified and suppressed in patients without GVHD or with mild GVHD by regulatory mechanisms. In the current study the patient had diarrhea, FTT, alopecia, erythroderma, and did not suffer from lymphadenopathy, hepatosplenomegaly and eosinophilia [3, 11].

Engrafted T cells may be CD4 or CD8, and, therefore, peripheral blood lymphocyte count may be normal or even elevated. Moreover, in the previous reports on SCID patients with prominent skin GVHD, the underlying variant was characterized by the absence of B cells. In the current study, the number of T-CD3 and T-CD4 is high, NK cells fall in normal range and B cells is only present in low levels [3, 6, 12].

Differentiation between GVHD and OS is often difficult in combined immunodeficiency with hypomorphic mutations only based on clinical and immunological phenotype [1]. Typical OS features can also be seen in some cases of maternally induced GVHD. Thus, some patients reported to have ‘Omenn’s syndrome’ may actually have had maternally-induced GVHD [13, 14]. In the current survey, the immunological and skin biopsies results of the patient were consistent with both GVHD and OS diseases. However, before confirmation of the maternal engraftment, the patient was evaluated based on the assumption that she was suffering from OS. The presence of maternal cells in the patient’s blood helped narrow the differential diagnosis in favor of microchimerism or GVHD.

Since the engrafted T cells do not have functional competence, the engraftment does not usually affect the course of the disease. However, a SCID patient with *JAK3* deficiency surviving to the age of 8 years with transplacentally acquired maternal T cells has been reported [11], suggesting the effect of these cells on the detectable immunologic functions. Our patient had some manifestations of GVHD, but she survived to the age of 4 years with normal levels of immunoglobulins. These residual immunologic functions may be due to maternally transferred T cells. The underlying cause is probably the HLA compatibility of the mother and the patient, due to parental consanguinity, which may have played an important role in the T, B, and antigen presenting cell collaboration. These data show that in rare cases, maternal engraftment may provide the required immune competence and lead to prolonged survival in the patients with SCID [11, 12].

Conventional techniques (e.g. Sanger sequencing), which are widely used in molecular diagnosis, cannot help to distinguish between haplotypes with different origins and to detect low-frequency alleles while massively parallel sequencing technologies provide now the possibility to study mutations at an appropriate level of resolution for detection of microchimerism and mosaicism [15]. In the current study, the WES analysis revealed the presence of microchimerism, PCR-cloning identifies low-frequency *JAK3* gene alleles [15, 16], and STR profiling (ASN-0002) helps detect origin of different cell lines in an SCID patient [17]. The results of these assessments confirmed the presence of a wild-type *JAK3* gene allele from maternal T cells in a ~ 10–20% population of the patient’s lymphocytes.

Other studies on at least 10 patients (particularly with mutations in the protein kinase 1 domain and normal T-cell subsets and NK cells) have also reported pathogenic variants in JAK3 gene leading to atypical SCID phenotypes because of the presence of maternal cells in the patients’ blood and bone marrow [12, 18, 19]. However, GVHD was not reported in the patients with hypomorphic mutations. Here we report a new hypomorphic mutation in an SCID patient with GVHD manifestation.

The next generation sequencing (NGS) Variant allele frequency (VAF) score reports are slightly different for the patients with microchimerism compared to genetically homogeneous patients with autosomal recessive/X-linked recessive mutations; therefore, analysis in this situation requires a more precise evaluation [20]. Special attention is to be paid to SCID patients to ensure that heterozygous alleles reported due to the misleading microchimerism status of the patients are not ignored.

In conclusion, it has been postulated that maternal T cells can prevent engraftment of bone marrow transplants from non-maternal donors, chiefly when HLA-haplocompatible T cell-depleted transplants are used. Because engraftment is incomplete, in most cases maternal cells fail to protect the host against infections. It is noteworthy that the proportion of patients presenting microchimerism with maternal lymphocytes may, in fact, be higher than that reported. It will be of interest to analyze SCID patients for detection of maternal cells by the use of even sensitive molecular techniques. Graft-versus-host disease due to maternal engraftment should always be suspected in a neonate with erythroderma.

## Supplementary information

**Additional file 1: Table S1.** PCR primers used for the PCR-sequencing of exon 17 of *JAK3* gene.

**Additional file 2.** Details of the metods used for Sanger sequencing, WES, T-A cloning and QF-PCR.

**Additional file 3.**  NGS variants data in VCF.7z format

**Additional file 4.** NGS variants data in VCF.txt format

**Additional file 5: Figure S1.** BAM file visualization by IGV (Integrative Genomic Viewer) software (https://software.broadinstitute.org).

**Additional file 6: Figure S2.** Sanger sequencing results of 10 plasmids containing different *JAK3* gene alleles. As shown one of these plasmids (P5) carries the wild type allele.

**Additional file 7: Figure S3.** Soup culture. White colonies had recombinant vector and were used for colony PCR and Sanger sequencing.

## Data Availability

Any additional data about the materials and methods will be provided by the corresponding authors upon request.

## Abbreviations

GVHD: graft versus host disease
SCID: severe combined immunodeficiency
STR: Short Tandem Repeat
QF-PCR: Quantitative Fluorescent Polymerase Chain Reaction
OS: Omenn syndrome
FTT: failure to thrive
HLA: human leucocyte antigen
WES: whole exome sequencing
ESID: European Society for Immunodeficiencies
ACMG: American College of Medical Genetics and Genomics
VAF: variant allele frequency

## References

[1] Bonilla FA, Khan DA, Ballas ZK, Chinen J, Frank MM, Hsu JT, Keller M, Kobrynski LJ, Komarow HD, Mazer B (2015). Practice parameter for the diagnosis and management of primary immunodeficiency. J Allergy Clin Immunol..

[2] Siala N, Azzabi O, Kebaier H, Mrad R, Barbouche R, Bejaoui M, Halioui S, Maherzi A (2014). Omenn Syndrome: two case reports. Acta Dermatovenerol Croat..

[3] Müller SM, Ege M, Pottharst A, Schulz AS, Schwarz K, Friedrich W (2001). Transplacentally acquired maternal T lymphocytes in severe combined immunodeficiency: a study of 121 patients. Blood.

[4] Al-Muhsen SZ (2010). Delayed presentation of severe combined immunodeficiency due to prolonged maternal T cell engraftment. Ann Saudi Med.

[5] Shulman Howard M., Kleiner David, Lee Stephanie J., Morton Thomas, Pavletic Steven Z., Farmer Evan, Moresi J. Margaret, Greenson Joel, Janin Anne, Martin Paul J., McDonald George, Flowers Mary E.D., Turner Maria, Atkinson Jane, Lefkowitch Jay, Washington M. Kay, Prieto Victor G., Kim Stella K., Argenyi Zsolt, Diwan A. Hafeez, Rashid Asif, Hiatt Kim, Couriel Dan, Schultz Kirk, Hymes Sharon, Vogelsang Georgia B. (2006). Histopathologic Diagnosis of Chronic Graft-versus-Host Disease: National Institutes of Health Consensus Development Project on Criteria for Clinical Trials in Chronic Graft-versus-Host Disease: II. Pathology Working Group Report. Biology of Blood and Marrow Transplantation.

[6] Denianke K, Frieden I, Cowan M, Williams M, McCalmont T (2001). Cutaneous manifestations of maternal engraftment in patients with severe combined immunodeficiency: a clinicopathologic study. Bone Marrow Transp.

[7] Abolhassani H, Kiaee F, Tavakol M, Chavoshzadeh Z, Mahdaviani SA, Momen T, Yazdani R, Azizi G, Habibi S, Gharagozlou M, Movahedi M, Hamidieh AA, Behniafard N, Nabavi M, Bemanian MH, Arshi S, Molatefi R, Sherkat R, Shirkani A, Amin R, Aleyasin S, Faridhosseini R, Jabbari-Azad F, Mohammadzadeh I, Ghaffari J, Shafiei A, Kalantari A, Mansouri M, Mesdaghi M, Babaie D, Ahanchian H, Khoshkhui M, Soheili H, Eslamian MH, Cheraghi T, Dabbaghzadeh A, Tavassoli M, Kalmarzi RN, Mortazavi SH, Kashef S, Esmaeilzadeh H, Tafaroji J, Khalili A, Zandieh F, Sadeghi-Shabestari M, Darougar S, Behmanesh F, Akbari H, Zandkarimi M, Abolnezhadian F, Fayezi A, Moghtaderi M, Ahmadiafshar A, Shakerian B, Sajedi V, Taghvaei B, Safari M, Heidarzadeh M, Ghalebaghi B, Fathi SM, Darabi B, Bazregari S, Bazargan N, Fallahpour M, Khayatzadeh A, Javahertrash N, Bashardoust B, Zamani M, Mohsenzadeh A, Ebrahimi S, Sharafian S, Vosughimotlagh A, Tafakoridelbari M, Rahimi M, Ashournia P, Razaghian A, Rezaei A, Mamishi S, Parvaneh N, Rezaei N, Hammarstrom L, Aghamohammadi A (2018). Fourth update on the Iranian national registry of primary immunodeficiencies: integration of molecular diagnosis. J Clin Immunol.

[8] Abolhassani H, Chou J, Bainter W, Platt CD, Tavassoli M, Momen T, Tavakol M, Eslamian MH, Gharagozlou M, Movahedi M, Ghadami M, Hamidieh AA, Azizi G, Yazdani R, Afarideh M, Ghajar A, Havaei A, Chavoshzadeh Z, Mahdaviani SA, Cheraghi T, Behniafard N, Amin R, Aleyasin S, Faridhosseini R, Jabbari-Azad F, Nabavi M, Bemanian MH, Arshi S, Molatefi R, Sherkat R, Mansouri M, Mesdaghi M, Babaie D, Mohammadzadeh I, Ghaffari J, Shafiei A, Kalantari N, Ahanchian H, Khoshkhui M, Soheili H, Dabbaghzadeh A, Shirkani A, Nasiri Kalmarzi R, Mortazavi SH, Tafaroji J, Khalili A, Mohammadi J, Negahdari B, Joghataei MT, Al-Ramadi BK, Picard C, Parvaneh N, Rezaei N, Chatila TA, Massaad MJ, Keles S, Hammarstrom L, Geha RS, Aghamohammadi A (2018). Clinical, immunologic, and genetic spectrum of 696 patients with combined immunodeficiency. J Allergy Clin Immunol..

[9] Alkhairy OK, Rezaei N, Graham RR, Abolhassani H, Borte S, Hultenby K, Wu C, Aghamohammadi A, Williams DA, Behrens TW, Hammarstrom L, Pan-Hammarstrom Q (2015). Rac2 loss-of-function mutation in 2 siblings with characteristics of common variable immunodeficiency. J Allergy Clin Immunol..

[10] Yao S, Hart DJ, An Y (2016). Recent advances in universal ta cloning methods for use in function studies. Protein Eng Des Sel.

[11] Tezcan I, Ersoy F, Sanal O, Turul T, Uckan D, Balci S, Hicsonmez G, Prieur M, Caillat-Zucmann S, Le Deist F (2005). Long-term survival in severe combined immune deficiency: the role of persistent maternal engraftment. J Pediatr.

[12] Palmer K, Green TD, Roberts JL, Sajaroff E, Cooney M, Parrott R, Chen D-F, Reinsmoen NL, Buckley RH (2007). Unusual clinical and immunologic manifestations of transplacentally acquired maternal T cells in severe combined immunodeficiency. J Allergy Clin Immunol..

[13] de Moraes RW, de Carvalho MHB, de Amorim-Filho AG, Francisco RPV, Romão RM, Levi JE, Zugaib M (2017). Validation of Qf-Pcr for prenatal diagnoses in a brazilian population. Clinics (Sao Paulo)..

[14] Appleton A, Curtis A, Wilkes J, Cant A (1994). Differentiation of materno-fetal Gvhd from Omenn’s syndrome in Pre-bmt patients with severe combined immunodeficiency. Bone Marrow Transplant.

[15] Wilbe M, Gudmundsson S, Johansson J, Ameur A, Stattin EL, Annerén G, Malmgren H, Frykholm C, Bondeson ML (2017). A novel approach using long-read sequencing and Ddpcr to investigate gonadal mosaicism and estimate recurrence risk in two families with developmental disorders. Prenat Diagn.

[16] Izawa K, Hijikata A, Tanaka N, Kawai T, Saito MK, Goldbach-Mansky R, Aksentijevich I, Yasumi T, Nakahata T, Heike T (2012). Detection of base substitution-type somatic mosaicism of the Nlrp3 gene with > 99.9% statistical confidence by massively parallel sequencing. DNA Res..

[17] Almeida JL, Cole KD, Plant AL (2016). Standards for cell line authentication and beyond. PLoS Biol.

[18] Cattaneo F, Recher M, Masneri S, Baxi SN, Fiorini C, Antonelli F, Wysocki CA, Calderon JG, Eibel H, Smith AR (2013). Hypomorphic janus kinase 3 mutations result in a spectrum of immune defects, including partial maternal T-cell engraftment. J Allergy Clin Immunol..

[19] Abolhassani H, Cheraghi T, Rezaei N, Aghamohammadi A, Hammarstrom L (2015). Common variable immunodeficiency or late-onset combined immunodeficiency: a new hypomorphic Jak3 patient and review of the literature. J Investig Allergol Clin Immunol.

[20] Strom SP (2016). Current practices and guidelines for clinical next-generation sequencing oncology testing. Cancer Biol Med..

