# Supplementary material for: Graft versus host disease and microchimerism in a JAK3 deficient patient
Source: Allergy Asthma Clin Immunol. 2019 Aug 22;15:47. doi: 10.1186/s13223-019-0361-2 (PMC6704686; doi:10.1186/s13223-019-0361-2)
Supplement: Supplementary file 1 — Additional file 1: Table S1. PCR primers used for the PCR-sequencing of exon 17 of JAK3 gene. [file 13223_2019_361_MOESM1_ESM.docx]

Table S1. PCR primers used for the PCR-sequencing of exon 17 of *JAK3* gene.

| **Primer name** | **Sequence (5’>3’)** |
| --- | --- |
| \| JAK3-F \| \| --- \| \| JAK3-R \| | \| AGCATGTCTGAGCAGTACCA \| \| --- \| \| CTTCCACTGGGCCCAATATG \| |
