# Supplementary material for: Graft versus host disease and microchimerism in a JAK3 deficient patient
Source: Allergy Asthma Clin Immunol. 2019 Aug 22;15:47. doi: 10.1186/s13223-019-0361-2 (PMC6704686; doi:10.1186/s13223-019-0361-2)
Supplement: Supplementary file 2 — Additional file 2. Details of the metods used for Sanger sequencing, WES, T-A cloning and QF-PCR. [file 13223_2019_361_MOESM2_ESM.docx]

# Additional data.

# Graft Versus Host Disease and Microchimerism in a JAK3-Deficient Patient

Shahbazi et al.

# Additional Methods

***Sanger Sequencing***

DNA was isolated from the whole blood by salting out. PCR runs with a final volume of 30 μl containing 100-200 ng genomic DNA, 10 pmol of each primers, and PCR master mix (Ampliqon). Initial denaturation of the genomic DNA was performed for 5 min at 94°C, followed by 30 cycles of amplification as follows: 30 s at 94°C, 30-45 s at 62°C and 60 s at 72°C. Evaluation of the PCR products was carried out with electrophoresis on a 1% agarose gel. Finally PCR products sequencing was carried out by the Macrogene Company. The results were analyzed using the Chromas and CLC workbench software, and evaluation of variants and report records check was carried out by online websites like NCBI, HGVS, HGMD, ENSEMBL and other databases.

***Whole Exome Sequencing***

Since over 13 genes are implicated in SCID and some of these genes are in omen syndrome, and regular molecular testing was not readily available for the genes, we resorted to whole exome sequencing. The sample was sent to the Macrogen company in Korea. The amount, concentration and optical density of the extracted DNA in this step were ≥3µgr, > 37.5 ng/µl and 1.8-2, respectively. Exome capture was performed on DNA using SureSelect V6-Post (IlluminaInc USA), and an average on-target coverage of over 25x on IlluminaHiSeq4000 (Illumina Inc.USA) was generated. Alignment was performed using BWA (v0.7.12-r1039)8, and variants calling and annotation was performed using GATK. For prioritization of the variants, we filtered all homozygous variants, further filtered by an allele frequency of <1% in the 1000Genome and ExAC. Variants in the 13 genes were prioritized and annotated for their deleteriousness using PredictSNP, MAPP, PhD-SNP, Polyphen1, Polyphen2, SIFT, and SNAP 11.

We found a missense mutation in *JAK3* gene, exon 17, c.2324G>A,CGT>CAT, p.R775H. This mutation was confirmed by the Sanger sequencing method, (see Table S1 for the primer sequences ). In the next step we used IGV software (v2.4.4) to visualize the mutation region. At this stage, we observed evidence of approximately 17% mosaicism or microchimerism.

***T-A Cloning***

To check the probability of mosaicism or microchimerism, we did T-A cloning with E.coli Top10 as the host, tetracycline resistance as the host selection marker, pGEM as the vector, ampicillin resistance as the vector transformation selection marker, PCR product as insert, and IPTG-XGAL as the selection marker for PCR product insertion into the vector. PCR product was cleaned up with Gene All Expin Combo GP kit ([GeneAll Biotechnology Co](https://www.google.com/url?sa=t&rct=j&q=&esrc=s&source=web&cd=2&cad=rja&uact=8&ved=0ahUKEwiHrOLvuKjZAhUDOhQKHUxWCosQFggwMAE&url=https%3A%2F%2Fwww.biotechzone.com%2Flisting%2Fchemicals-reagents%2Fexpin-pcr-sv-50-prep%2F&usg=AOvVaw1VA2eNgADzS8c4hJKkbA1J)). Ligation of the vector and PCR product was done using the ligase enzyme (NEB Co.), its buffer, vector, insert, water, and over-night incubation in 4C. In the transformation step, we used CaCl2 and heat shock to make the host competent. After the transformation step, we cultivated the bacteria that were supposedly transformed in the LB agar plates containing Amp and IPTG-Xgal. Afterward, we selected the white colonies that had recombinant vector and did colony-PCR. The results confirm transformation success. These recombinant plasmids were extracted using [Plasmid DNA Purification Kit](https://www.qiagen.com/us/shop/sample-technologies/dna/plasmid-dna/qiagen-plasmid-kits/) (Qiagen co.) and then sent to the Macrogen co. for Sanger sequencing with the universal primers T7-F and SP6-R **.**

### *QF-PCR*

In order to find the origin of these different cell lines in the patient, we did QF-PCR for trisomy STR-markers, in Kawsar Biotechnology co. using of the Applied BiosystemGenetic Analyzer and GeneMaper 4.1 software. The results showed some peaks and noises which looked like chorionic villus sample contamination with maternal cells.
