# Supplementary figures and images for: Graft versus host disease and microchimerism in a JAK3 deficient patient
Source: Allergy Asthma Clin Immunol. 2019 Aug 22;15:47. doi: 10.1186/s13223-019-0361-2 (PMC6704686; doi:10.1186/s13223-019-0361-2)

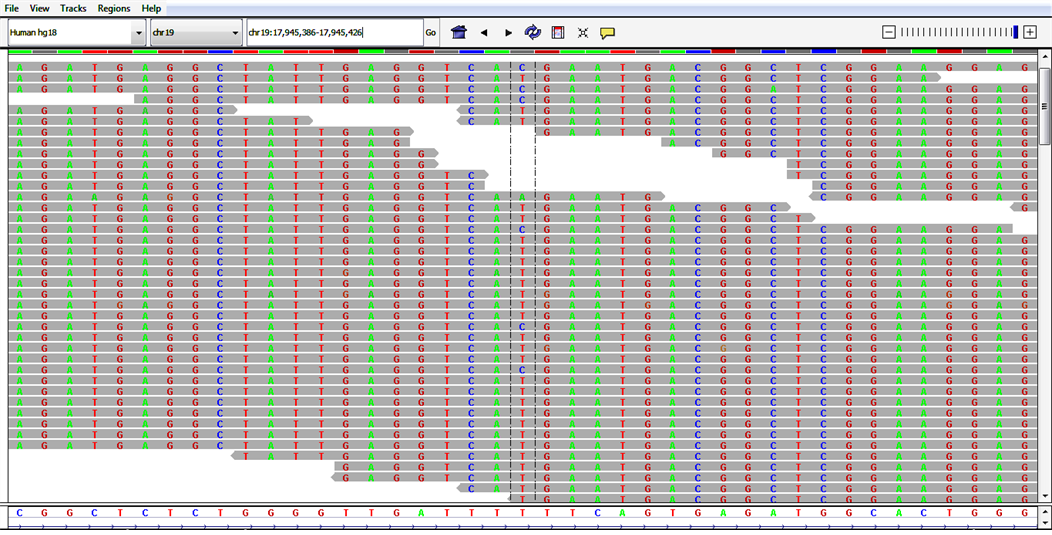

Supplement: Supplementary file 5 — Additional file 5: Figure S1. BAM file visualization by IGV (Integrative Genomic Viewer) software (https://software.broadinstitute.org). [file 13223_2019_361_MOESM5_ESM.png]

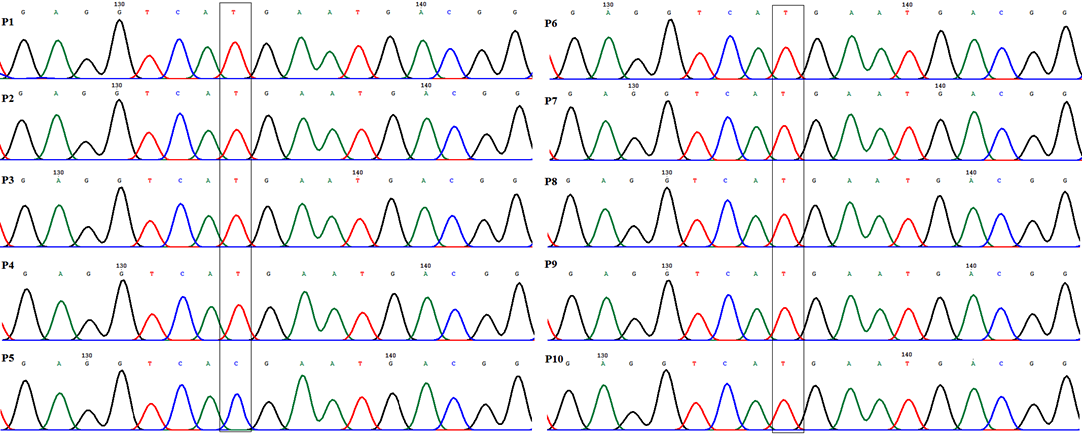

Supplement: Supplementary file 6 — Additional file 6: Figure S2. Sanger sequencing results of 10 plasmids containing different JAK3 gene alleles. As shown one of these plasmids (P5) carries the wild type allele. [file 13223_2019_361_MOESM6_ESM.png]

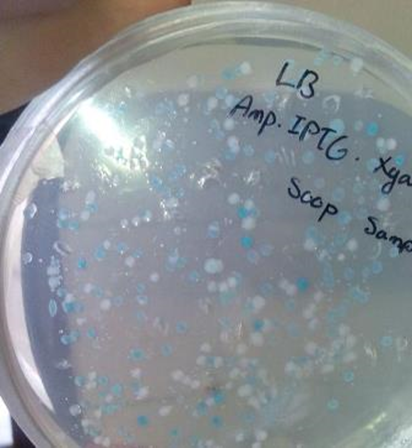

Supplement: Supplementary file 7 — Additional file 7: Figure S3. Soup culture. White colonies had recombinant vector and were used for colony PCR and Sanger sequencing. [file 13223_2019_361_MOESM7_ESM.png]
